# Prediction of conversion from mild cognitive impairment to Alzheimer’s disease and simultaneous feature selection and grouping using Medicaid claim data

**DOI:** 10.1186/s13195-024-01421-y

**Published:** 2024-03-09

**Authors:** Qi Zhang, Ron Coury, Wenlong Tang

**Affiliations:** 1 Department of Mathematics and Statistics, University of New Hampshire, Durham, NH 03824 USA; 2 Takeda Pharmaceuticals, Cambridge, MA 02142 USA

**Keywords:** Alzheimer’s disease (AD), Mild cognitive impairment (MCI), Machine learning, Real-world data, Feature selection, Feature grouping

## Abstract

**Background:**

Due to the heterogeneity among patients with Mild Cognitive Impairment (MCI), it is critical to predict their risk of converting to Alzheimer’s disease (AD) early using routinely collected real-world data such as the electronic health record data or administrative claim data.

**Methods:**

The study used MarketScan Multi-State Medicaid data to construct a cohort of MCI patients. Logistic regression with tree-guided lasso regularization (TGL) was proposed to select important features and predict the risk of converting to AD. A subsampling-based technique was used to extract robust groups of predictive features. Predictive models including logistic regression, generalized random forest, and artificial neural network were trained using the extracted features.

**Results:**

The proposed TGL workflow selected feature groups that were robust, highly interpretable, and consistent with existing literature. The predictive models using TGL selected features demonstrated higher prediction accuracy than the models using all features or features selected using other methods.

**Conclusions:**

The identified feature groups provide insights into the progression from MCI to AD and can potentially improve risk prediction in clinical practice and trial recruitment.

**Supplementary Information:**

The online version contains supplementary material available at 10.1186/s13195-024-01421-y.

## Background

Alzheimer's disease (AD) is a progressive neurodegenerative disorder that leads to memory impairment, behavioral changes, and other cognitive function deficits. There were no promising disease modifying therapies for AD until the approval of aducanumaband and lecanemab. The availability of these drugs is currently limited, and they demonstrated better effectiveness in patients at the early stages of the disease compared to the late stage. Therefore, it remains crucial to identify high-risk individuals early and intervene based on modifiable risk factors.

Risk stratification is also vital for guiding future trials for AD. Clinical trials, particularly Phase III trials, are known for their high costs. Those for neurodegenerative diseases like AD face additional challenges due to the potentially slow disease progression in some subjects and the limited duration of the trials (typically one to three years), which may not capture treatment effects, even if they exist. As a result, at least 98% of AD trials have failed [1]. Even successful trials such as those for aducanumab and lecanemab are not without limitations and controversies. Since diagnosing AD is a complex and potentially lengthy process, recent trials measured treatment effects based on differences in certain cognitive tests, biomarkers such as amyloid, or composite outcome measures within the trial timeframe. By developing a model that identifies a sub-population of early-stage AD patients at a higher risk of progressing to the late stage within the common trial timeframe, future trials can be designed to target this specific population and utilize more definitive primary outcomes such as the diagnosis of late-stage AD.

A relevant problem in this context is predicting the conversion from Mild Cognitive Impairment (MCI) to dementia due to AD. MCI serves as an intermediate stage between preclinical Alzheimer's disease and dementia due to Alzheimer's, representing a natural entry point into the Alzheimer's and dementia care system. Depending on the study population, about 10.2 to 33.6% MCI patients proceed to AD annually, while others either experience no further cognitive decline or return to normal cognition [2]. Such heterogeneity is partially due to the broader definition of MCI, and poses a similar treatment challenge, making it critical to identify MCI patients at high risk of conversion to AD through predictive modeling.

In the literature, numerous risk-prediction models have been developed for different target populations and dementia-related outcomes, utilizing various data types. See Chen et al. (2022) [3] for a review of the prediction models for conversion from MCI to AD. Many of these models incorporate brain imaging, cerebrospinal fluid analysis, genetic variants, and dementia-specific tests like neuropsychological test scores. Some studies have taken advantage of the rich resources provided by the ADNI project [4] to improve the prediction of conversion from MCI to AD by combining genetics, quantitative brain magnetic resonance imaging, and cognitive measures into a single model [5]. While these studies have achieved encouraging prediction performance, they fall short in addressing the needs of clinical practice and recruitment for clinical trials due to the non-routine collection of these data in clinical care settings. Over the past five years, an increasing number of works on AD risk prediction have emerged, leveraging routinely collected electronic health record (EHR) and administrative claim data. For example, machine learning models have been developed to predict AD among the seniors without prior dementia using the carefully chosen features from administrative claim data [6, 7], or pre-aggregated features constructed from EHR data [8, 9].

To the best of our knowledge, our study is the first that predicts the conversion from MCI to AD using routinely collected administrative claim data, addressing a critical gap in the existing literature. Additionally, we are interested in identifying the features that play a crucial role in this conversion process. Instead of pre-aggregating and aggressively filtering features to reduce dimensionality, as commonly done in previous studies, we propose a novel data-adaptive procedure that simultaneously predicts MCI to AD conversion and selects and groups the most important features.

## Methods

### Data and study cohorts

The study utilized MarketScan Multi-State Medicaid data in the OHDSI Observational Medical Outcomes Partnership (OMOP) model, which was provided by International Business Machines (IBM) Corporation. The IBM MarketScan databases are constructed by collecting data from employers, health plans and state Medicaid agencies. The data encompass service-level claims for inpatient and outpatient services as well as outpatient prescription drugs. The database is designed to provide long-term longitudinal observational data and includes 32.87 million patients.

**Fig. 1:**
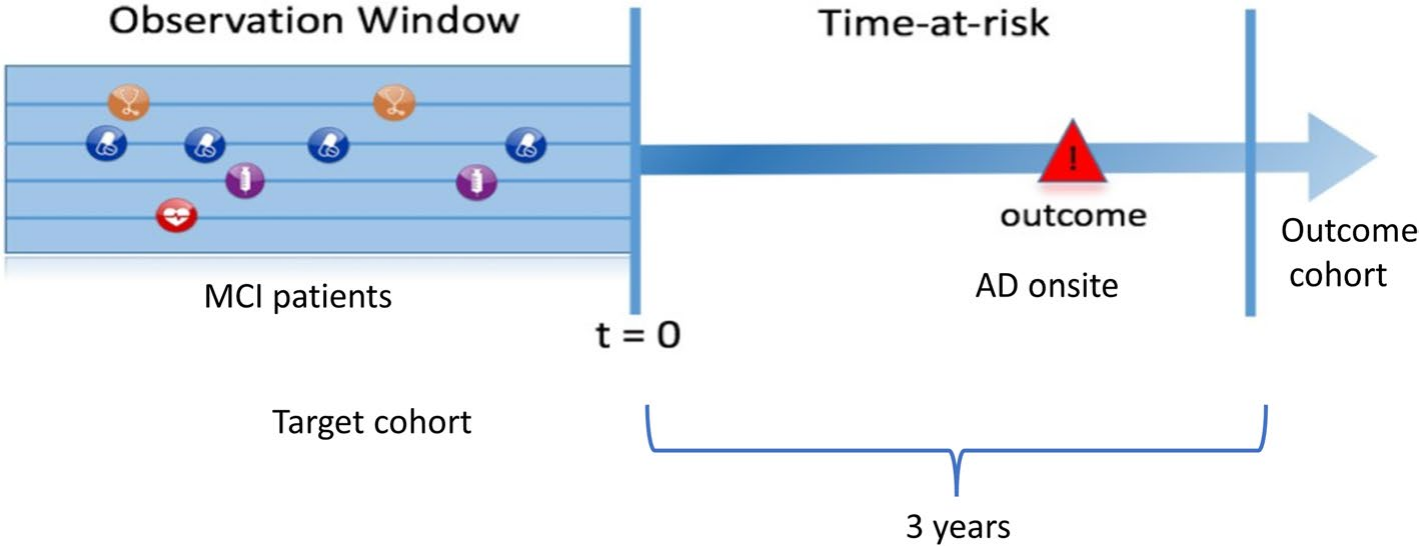
Setup of the cohort

A Cohort study was designed to investigate patients diagnosed with Mild Cognitive Impairment (MCI) in 2006-2016. The aim was to predict their conversion to AD within 3 years after the MCI diagnosis using their administrative claim history during the one-year observation window prior to the MCI diagnosis (Fig. 1). AD and MCI were defined using International Classification of Diseases, Ninth/Tenth Revision (ICD9/ICD10) diagnosis codes (Table 1). Since AD is an age-related disease, the focus was on patients 50 years of age or older at the time of their MCI diagnosis. Patients with medical history less than a year prior to the MCI diagnosis were excluded, as were patients with less than 3 years of follow-up after the MCI diagnosis. This leads to 6,847 patients, among whom 312 converted to AD within 3 years. Stratified sampling based on whether being converted to AD within 3 years was used to select 70% of the patients as the training cohort, while the remaining 30% of the patients formed the validation cohort. All variable selection and machine learning model training were done using the training cohort alone, and the validation cohort was only used for evaluation.

**Table 1:** ICD9/ICD10 diagnosis codes for MCI and AD

Code	Name	Class	Vocabulary
G31.84	Mild cognitive impairment, so stated	5-char billing code	ICD10CM
331.83	Mild cognitive impairment, so stated	5-dig billing code	ICD9CM
G30.8	Other Alzheimer disease	ICD10 code	ICD10
G30.9	Alzheimer disease, unspecified	ICD10 code	ICD10
G30.0	Alzheimer disease with early onset	ICD10 code	ICD10
G30.1	Alzheimer disease with late onset	ICD10 code	ICD10
G30.8	Other Alzheimer's disease	4-char billing code	ICD10CM
G30.1	Alzheimer's disease with late onset	4-char billing code	ICD10CM
G30.0	Alzheimer's disease with early onset	4-char billing code	ICD10CM
G30	Alzheimer's disease	3-char non-bill code	ICD10CM

Most covariates were binary variables for the ICD codes for the administrative claims. Duplicated features and features with no incidence were removed, resulting in 16,862 remaining rare features. Additionally, 11 baseline variables were included in the study: CHADS2, CHADS2VASc, gender, race (White and Black or African American), ethnicity (Hispanic or Latino), and the linear, quadratic, and cubic terms of age at MCI diagnosis. CHADS2 and CHADS2VASc scores were two scores for atrial fibrillation stroke risk, and we scaled them to a range of 0 to 1. Patient age was normalized by centering it at 65 years old and then dividing it by 20.

### Tree-guided lasso for Rare feature selection and aggregation

Consider a logistic regression model where *Y*_*i*_~*Bernoulli*(*p*_*i*_) and *logit*(*p*_*i*_) = *X*_*i*_*β* for i=1, … ,n. Here *Y*_*i*_ is the indicator of whether the patient progressed to AD, *X*_*i*_ is a length p feature vector from the health claim dataset for subject i, β is the length p regression coefficient vector. Let X be the n by p feature matrix that includes all n subjects in the data, where the ith row corresponds to *X*_*i*_. This feature matrix, derived from Administrative Claim data, primarily consists of an incidence matrix of ICD10 codes, with most entries being 0. In statistical literature, such features are sometimes referred to as “rare features” [10]. Rare features usually lack sufficient variation among samples to effectively measure their association with the outcome. Consequently, these features are intentionally discarded during data preprocessing or implicitly during model selection based on how the algorithm prioritizes the features. The scientific community has recognized this issue, and one approach to mitigate it involves pre-clustering the features and aggregating the rare features within the same group based on previous studies [11]. However, even after aggregation, these features may still be too rare for subsequent variable selection, necessitating further ad hoc feature aggregation. This is partially due to the separation of the feature aggregation step from variable selection, which fails to adapt to the importance of these features. Combining these two steps could potentially enhance variable selection performance and enable identified clusters to better adapt to their importance in the regression model.

In practice, rare features are often related through a tree structure. For instance, the phylogenetic tree among microbiome species can be used to link the microbiome features. In general, this tree can be learned through hierarchical clustering of the data. Based on this tree, the effect of each leaf (elements of β) can be decomposed as the effects of their ancestors plus a leaf-specific effect. Let Γ be a tree with leaves1, … ,p, and the effect of node *u* ∈ *Γ* is *γ*_*u*_. For j=1, … ,p, *β*_*j*_ can be expressed as ${\beta}_j=\sum_{u\in ancestor(j)\cup \left\{j\right\}}{\gamma}_u$ where *ancestor*(*j*) denotes the indices of all the ancestor internal nodes of the terminal node j based on the tree. Let q be the number of nodes in the tree Γ, and A be a p-by-q binary matrix such that *A*_*ju*_ = 1 if *u* ∈ *ancestor*(*j*) ∪ {*j*} and 0 otherwise. There is *β* = *Aγ*.

We propose transforming the feature matrix to $\overset{\sim }{X}= XA$. Consequently, the systematic component of the logistic regression becomes $logit\left({p}_i\right)={\overset{\sim }{X}}_i\gamma$. We adopt the weighted lasso penalty $\sum_{u\in \varGamma }{q}_u^{1/2}\mid {\gamma}_u\mid$ where *q*_*u*_ is the number of leaves that are the children of node u. Let $\hat{\gamma}$ be the estimate, and the estimate of β, the regression coefficients for the effects of the original features, is $\hat{\beta}=A\hat{\gamma}$. As this estimate is guided by the tree, we refer to this method as “tree-guided lasso” (TGL). There is only one sparsity penalty parameter, which is selected by 5-fold cross-validation. The output of this model includes the important groups of features and the regression coefficients.

### Co-occurrence tree for ICD10 codes

To define the tree of features using the data, we first establish the distance matrix D among the features based on the co-occurrence of rare features. For two features j and k, Let *n*_*j*_ and *n*_*k*_ denote their frequencies in the sample, respectively. Furthermore, let *n*_*jk*_ be the number of times they co-occur. We define their distance ${D}_{jk}=1-\frac{n_{jk}}{\sqrt{n_j{n}_k}}$. Hierarchical clustering with average agglomeration is then applied to this distance matrix to build the tree, which guides the tree-guided lasso algorithm mentioned earlier. To avoid large clusters, we remove internal nodes with a large number of descendants, retaining only the treelets with no more than 50 leaves.

### Tight-clustering for extracting important groups of features

High dimensional variable selection and clustering are both notoriously difficult and may be sensitive to the randomness of the data. The rareness of the feature matrix further exacerbates these problems. Averaging the results from repetitive subsamples has been applied to improve the finite sample performance of variable selection [12] and extracting meaningful tight clusters from the data [13]. We employ similar techniques to extract robust clusters of important rare features.

**Fig. 2:**
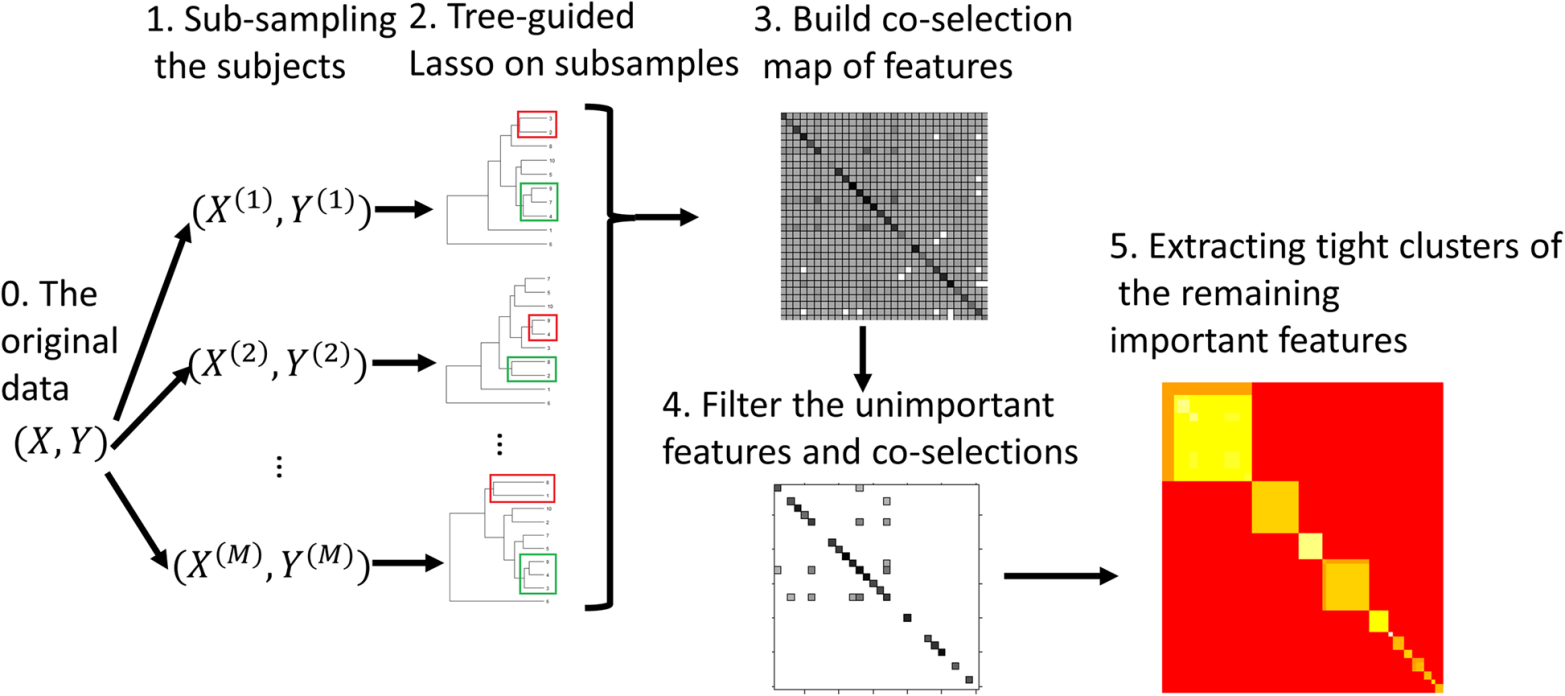
The tree-guided lasso (TGL) workflow: it aggregates the tree-guided lasso outputs from repetitive sub-samples and outputs important feature groups

As depicted in Fig. 2, for each replication, we randomly subset 80% of the training data, discard the extremely rare features (frequency <*n*^0.2^,e.g., at least 7 incidences out of 10,000 samples), build the tree, and employ the tree-guided lasso. We create a co-selection matrix among the features to summarize the results across B=100 replications. Each element in the ith row and jth column of this matrix represents the number of replicates in which the ith and jth features are both selected and grouped together (with a common ancestor node with nonzero effect). We further filter the features, excluding those with a selection proportion less than *π*_*imp*_ . Additionally, elements in the co-selection matrix below *π*_*co*_ are set to zero. Both *π*_*imp*_ and *π*_*co*_ are user-defined tuning parameters for interpretation purpose. We use 0.5 for both in our analysis. The remaining features are clustered into disjoint groups based on this co-selection map. The output of this TGL workflow in Fig. 2 includes robust groups of important features for predicting the conversion from MCI to AD.

### Predictive modeling using the extracted features

We train various predictive models on the training cohort data using the extracted features from the proposed feature selection workflow described in Section 2.4. The selected features within the same group are aggregated using three strategies. The first strategy involves using them as individual features. The second strategy sums up the features in the same group. The third approach leverages the binary nature of the original features, which represent the incidence of ICD10 codes, and defines the aggregated feature for the group as 1 if any of the original features in the group is 1, and 0 otherwise. The predictive models used include logistic regression (LR), generalized random forest (GRF) [14], and artificial neural network (ANN) [15]. We train these models on the training data using [1] all features, [2] Lasso selected features, [3] GRF selected features, [4] TGL selected features, [5] group sums of the TGL selected features, and [6] group unions of the TGL selected features. GRF ranks the variables by importance, and a cutoff is chosen so that it selects the same number of features as TGL. When training logistic regression on all features, we apply the lasso penalty with the tuning parameter lambda selected via 5-fold cross-validation. For GRF, we train it on the training data with the default settings, except that we average 10,000 trees and assign re-sampling weights for the cases proportional to the inverse of the events' prevalence. The ANN architecture consists of two hidden dense layers with 64 nodes and ReLu activation function. The output layer has one node and Sigmoid activation function. We determine the number of epochs via 5-fold cross-validation. All feature selection procedures and the predictive models are trained using the training cohort, and their performance is evaluated on the validation cohort.

### Model evaluation

The proportion of conversion to AD from MCI is lower than 6%, making it an imbalanced classification problem. The Precision-Recall Curve (PRC) is a better measure of performance than the ROC curve in such cases [16]. We can also calculate the Area Under the PRC (AUPRC). While the Area Under the ROC (AUROC) for a useless random classifier is 0.5, its corresponding AUPRC is equal to the proportion of true cases in the data (AD conversion rate in our context). Extremely unbalanced classification problems like ours often yield small AUPRC values, such as 0.1.

For the cross-validation step in tree-guided lasso, AUPRC is maximized. For the evaluation of the predicted models based on all or the selected features, the models are trained using the training cohort, and both AUPRC and AUROC are calculated on the validation cohort. R package pROC is used to calculate AUROC and its confidence interval is based on the “delong” method. AUPRC is calculated using R package PRROC, and the confidence interval is based on the Logit interval proposed in Boyd et al (2013) [17].

### Relative risk for the important feature groups

The relative risks and the associated 95% confidence intervals for the important feature groups identified by TGL are calculated for the validation cohort. The features in each group are aggregated as one feature using the group sum as described in Section 2.5. The exposure and the non-exposure groups are defined based on whether the aggregated feature exceeds its median value. Due to the strong association between age and the other variables, the relative risks for features other than age are stratified by age group (50-59,60-69,70-79, and above 80). For each aggregated feature, a point estimate and a confidence interval are calculated by inverting a score test statistics for its average stratified relative risk (equation 6 of Tang 2020 [18]).

## Results

### Dataset characteristics

The training and validation cohorts consist of 6,847 MCI patients in total, with 312 of them converting to AD within 3 years. The conversion rate is approximately 4.56%. The average age at the time of MCI diagnosis for those who later converted to AD was 71.8 years, while it was 66.2 years for those who did not convert. As expected, age was found to be a significant factor in our analysis in the next section. Among the subjects, 67.6% are female, 53.0% are White, 29.5% are Black or African American, and 1.2% identify as Hispanic. Most of the covariates are incidences of ICD codes, and the majority of their entries are 0s. After removing features with all 0s, the median incidence rate is 0.88% (roughly 6 occurrences out of 6,847 MCI patients). Consequently, the feature matrix consists primarily of rare features. The data distributions of the training cohort and the validation cohort are similar (Table S1). However, we acknowledge that the distributions of many of the individual codes in the two cohorts can be different due to the rarity and the high dimensionality of the data alone.

### Variable selection

The proposed tree-guided lasso (TGL) workflow as depicted in Fig. 2 was repeated 100 times.

**Fig. 3:**
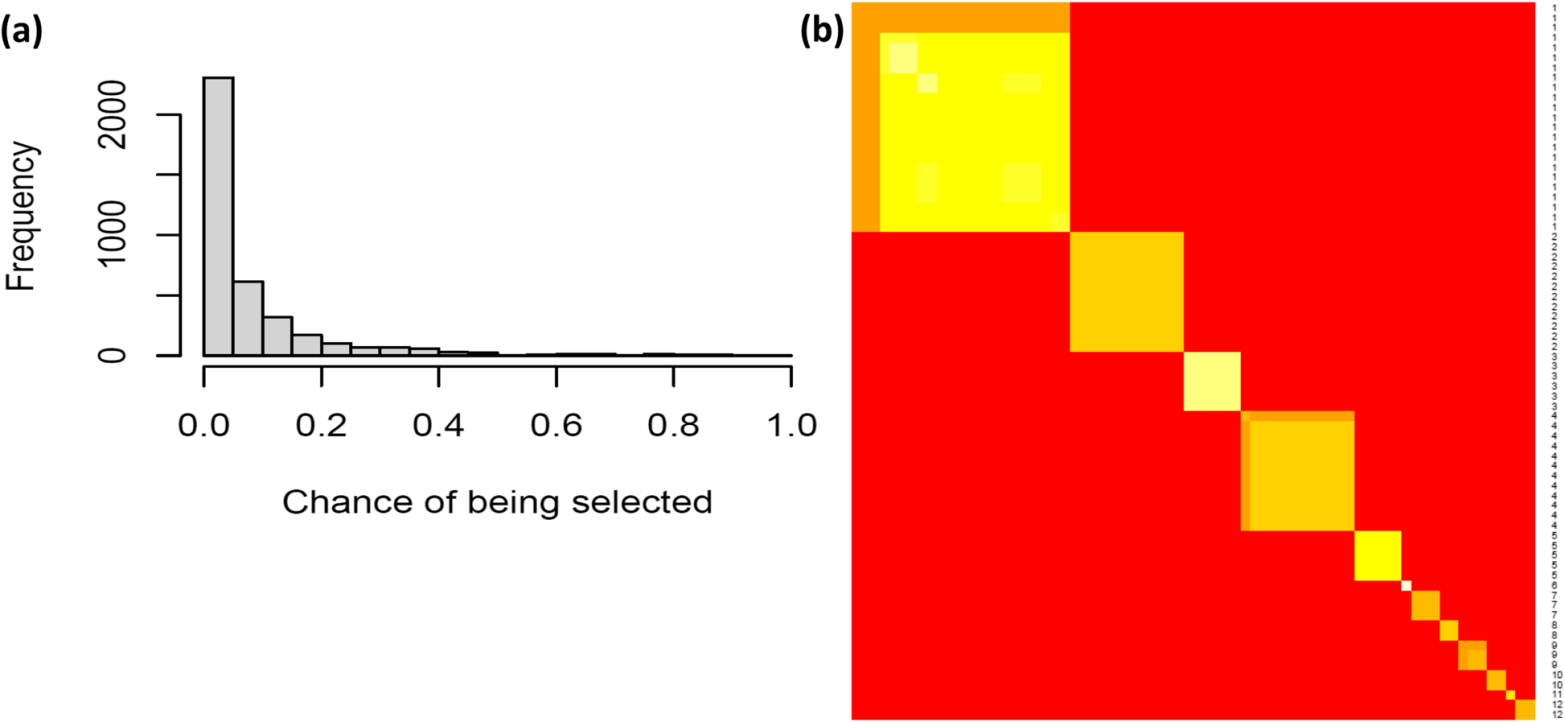
**a** Histogram of the numbers of sub-sampling replicates that each covariate is selected as an important covariate, conditional on that it is selected in any replicates. **b** Co-occurrence map of the 72 selected important covariates, grouped in 12 perfect tight clusters

Out of the 16,862 covariates, 3,823 of them were selected as important covariates by TGL in at least one of the 100 replications. Fig 3a illustrates the frequency of selection for these variables in the sub-sampling replicates. It was observed that most covariates were selected in only a few sub-sampling replicates, indicating that their association to the response are likely to be spurious due to the randomness of the sub-sampling process. The truly important covariates are those that were frequently selected.

There were 72 covariates selected in at least 50% of the sub-sampling replicates, forming 12 distinct clusters that were completely disjointed from each other (Fig. 3b). Additional file 1 presents these covariates, their importance measured by the relative frequency of selection, and their cluster assignments. Most of these clusters have clear interpretations (Table 2). For instance, cluster 3 includes 6 covariates related to Apnea, ranking as the second most important cluster after age. It is also the only cluster with significant relative risk after stratified by age. Cluster 4 consists of 12 covariates associated with viral diseases of the abdomen, such as viral hepatitis C. The 5 covariates in Cluster 5 are related to intervertebral issues. Cluster 1, the largest and most heterogeneous cluster, comprises 23 covariates of varying importance. These covariates are largely associated with the intake of pain medication. The two clusters with only one covariate are the linear term of age and Opioid. We remark that the relative risk for Cluster 8 cannot be calculated because there is no conversion in the low-risk group (138 out of 2055), and the Cluster 9 and 11 are combined based on their similar interpretation for robust calculation of the relative risk.

**Table 2:** The interpretation, the number of covariates and the relative risk of the important feature clusters calculated using the validation cohort. The specific covariate names are in Additional file 1

Cluster ID	Cluster Interpretation	Number of Covariates	Relative Risk (95% CI)
1	Pain medicine and acid disorder medication	23	0.75 (0.35, 1.14)
2	Electrolyte solution intake	12	1.02 (0.49, 1.55)
3	Apnea	6	0.53 (0.10, 0.95)
4	Infection of Abdomen such as Heptatitis C	12	0.56 (0.01, 1.09)
5	Intervertebral issues	5	1.06 (0.53, 1.59)
6	Age	1	3.19 (2.01, 5.00)
7	Joint Pain	3	0.68 (0.14, 1.23)
8	Diagnosis of cognitive disorder before MCI	2	NA
9	Strong painkillers such as oxycodone	3	0.83 (0.37,1.30)
11	Opioids	1	
10	Brain injury or lesion	2	1.09 (0.55, 1.62)
12	Senile dementia	2	1.56 (0.69, 2.43)

### Prediction using the extracted features

We assessed how the extracted features influenced predictive modeling by evaluating their performance on the validation cohort (Table 3). Using the selected features improved the predictive capability of logistic regression. Logistic regression using the TGL selected features without aggregation yielded the highest AUPRC of 0.098 and the highest AUROC of 0.726 with aggregation by group unions, compared to 0.079 and 0.682 when using all features, and 0.077 and 0.684 when using the features pre-selected by regular lasso. We also considered the feature set selected by a nonlinear model GRF. To our surprise, the nonlinear predictive model GRF does not perform well regardless of whether using all features or the pre-selected features based on an initial GRF fit. Other combinations of feature sets and predictive models are also explored. Most differences in AUPRC are not statistically significant based on the 95% confidence intervals. The AUROC values for models trained using all features, Lasso features or GRF features are generally close to or lower than the lower bound of the 95% CI of the AUROC for the logistic regression model based on the group union of TGL features.

**Table 3:** The predictive performance (AUPRC and AUROC with 95% CI) of ML models constructed using all features or only the selected features

		Logistic regression	GRF	ANN
All features	AUPRC	0.079 (0.038,0.153)	0.072 (0.034,0.145)	0.052 (0.022, 0.120)
	AUROC	0.681 (0.632,0.731)	0.686 (0.642,0.730)	0.576 (0.524,0.628)
Lasso features	AUPRC	0.077 (0.038,0.151)	0.080 (0.040, 0.155)	0.070 (0.033,0.143)
	AUROC	0.684 (0.636,0.733)	0.682 (0.631,0.732)	0.671 (0.624,0.719)
GRF features (72)	AUPRC	0.075 (0.037,0.149)	0.075 (0.036, 0.148)	0.067 (0.031,0.139)
	AUROC	0.679 (0.629,0.729)	0.696 (0.651,0.741)	0.654 (0.603,0.705)
TGL features	AUPRC	0.098 (0.052,0.176)	0.077 (0.038,0.151)	0.076 (0.037,0.150)
	AUROC	0.709 (0.658,0.760)	0.701 (0.657,0.744)	0.682 (0.634,0.730)
Group sum of TGL features	AUPRC	0.091 (0.049,0.169)	0.074 (0.036,0.148)	0.078 (0.038,0.152)
	AUROC	0.723 (0.678,0.768)	0.695 (0.652,0.738)	0.692 (0.646,0.737)
Group union of TGL features	AUPRC	0.094 (0.049,0.172)	0.074 (0.036,0.148)	0.086 (0.044,0.163)
	AUROC	0.726 (0.681,0.771)	0.693 (0.647,0.738)	0.712 (0.665,0.758)

## Discussion

In this paper, we addressed the problem of predicting the conversion from MCI to AD using administrative claim data. Our approach involved developing a novel machine learning model that simultaneously predicted the conversion to AD from MCI and grouped the important features. The proposed model is an example of sparse regularization for high dimensional data that exploits the structured sparsity such as tree structure [10, 19–21].In particular, Kim and Xing (2012) [19] developed tree-guided group lasso for multiple response regression in which the multiple responses are related based on a tree. In contrast, our model exploited the tree structure among the predictors. This is similar to Yan and Bien (2021) [10]. However, their algorithm cannot be applied to classification, while ours can. Our proposed pipeline, combining this novel model and repetitive sub-sampling, resulted in robust groups of predictive features for the conversion from MCI to AD.

Many of the identified feature groups have well-documented associations with AD and related dementia in the existing literature. The feature with the highest importance measure is age (Cluster 6). In our context, senile dementia (Cluster 12), an outdated term, is essentially an indicator variable that the patient was older than 65 when diagnosed with MCI, suggesting that the age at onset of MCI impacts the progression towards AD.

The cluster with the second highest average importance comprises six features related to apnea (Cluster 3). The association between cognitive decline and apnea has been extensively documented in the literature and confirmed through meta-analysis [22]. This finding is particularly interesting because apnea is a reversible condition. Numerous studies have investigated whether treatment of obstructive sleep apnea (OSA) with continuous positive airway pressure (CPAP) in patients with cognitive impairment, many of which have concluded that CPAP does have an effect [23]. In our analysis, this feature cluster presents a marginally lower risk of conversion, which may be the effect of apnea treatment. It will be interesting to study the causal effects of apnea treatment on preventing AD using a larger dataset.

The largest cluster (Cluster 1) comprises 23 drug codes for various conditions, many of which are used for pain management. Three other important clusters are opioids (Cluster 11), opioid derivatives (Cluster 9), and joint pain (Cluster 7). Long-term Opioids use is generally related to increased risk of dementia [24].

There is a cluster of five features related to the degeneration of spine (Cluster 5). It has been found that there was a strong association between spondylosis (up to 15 years before diagnosis) and AD risk, even after accounting for other identified risk factors [25]. It was argued that this association was not solely caused by the inflammatory nature of spondylitis. At the molecular level, the literature suggests that intervertebral disc degeneration and tau protein hyperphosphorylation are both regulated through the AMPK/GSK3β pathway [26, 27].

The other important clusters also exhibit interpretable connections to AD. Cluster 10 consists of brain lesions and injuries. The association between brain injury or brain lesions and AD has been well-documented in the literature [28, 29]. Cluster 8 (diagnosis of cognitive disorder before MCI) suggests that these patients may have experienced MCI onset before their formal diagnosis, highlighting the complexity of accurately diagnosing such conditions. Cluster 4 represents infectious diseases of the abdomen, such as Hepatitis C, with an importance measure of 0.61. The association between AD and viral infections of the abdomen, such as Hepatitis C, has long been debated [30–34]. Our results support the existence of an association, although the causality of such an association remains unclear. Cluster 2 comprises covariates representing the prescription of electrolyte solutions such as sodium chloride. The mechanism behind its association with AD is currently unknown.

We re-trained machine learning models using the TGL selected features, and their prediction accuracy on the validation cohort was better than the same modeled trained using all features or the selected features based on other methods. This finding highlights the potential of using the proposed TGL workflow and administrative claim data to examine the heterogeneity in the risk of AD among MCI patients. Furthermore, combining EHR data and claim data may further improve accuracy.

However, this study has limitations. Firstly, the sub-population covered by Medicaid claim data may differ from those covered by other types of medical insurances. Secondly, both MCI and AD diagnoses are complex, and many positive cases may go unreported in our dataset.

## Conclusions

In conclusion, we demonstrate the potential to utilize routinely collected administrative claim data for predicting the conversion from MCI to AD. Through a purely data-driven approach, we successfully identify and group important features simultaneously. These feature groups are interpretable and align largely with findings in the existing literature. However, it is important to note that administrative claim data, while powerful, may not capture all the information within patients' medical history. Nevertheless, our approach can serve as a starting point for future research aimed at combining multiple sources of routinely collected health history data to predict dementia-related diseases and their progression.

### Supplementary Information

**Supplementary Material 1.**

**Supplementary Material 2.**

## Data Availability

The data that support the findings of this study are available from IBM MarketScan databases but restrictions apply to the availability of these data, which were used under license for the current study, and so are not publicly available.

## Abbreviations

AD: Alzheimer’s disease
MCI: Mild cognitive impairment
EHR: Electronic health record
TGL: Tree-guided lasso
ANN: Artificial neural network
GRF: Generalized random forest precision-recall curve
PRC: Precision-recall curve
AUPRC: Area under precision-recall curve
ROC: Receiver operating characteristic
AUROC: Area under receiver operating characteristic curve
