# Supplementary material for: Prediction of conversion from mild cognitive impairment to Alzheimer’s disease and simultaneous feature selection and grouping using Medicaid claim data
Source: Alzheimers Res Ther. 2024 Mar 9;16:54. doi: 10.1186/s13195-024-01421-y (PMC10924319; doi:10.1186/s13195-024-01421-y)
Supplement: Supplementary file 2 — Supplementary Material 2. [file 13195_2024_1421_MOESM2_ESM.docx]

**Table S1:** Characteristic comparison of the training cohort and the validation cohort.

outcome variables, main risk variables, age, gender, race, proportion of converters, and time to conversion .

|  | Training cohort | Validation cohort |
| --- | --- | --- |
| Conversion rate | 0.045 | 0.046 |
| Average age | 66.6 | 66.4 |
| Proportion of male | 0.32 | 0.32 |
| Proportion of Black or African American | 0.30 | 0.29 |
| Proportion of White | 0.53 | 0.53 |
| Average of Charlson index -Romano adaptation | 0.23 | 0.22 |
| Average of Diabetes Comorbidity Severity Index (DCSI) | 0.37 | 0.36 |
| Average of the normalized CHADS2 index | 0.48 | 0.47 |
| Average of the normalized CHADS2VASc index | 0.45 | 0.45 |
| Median number of incidences of ICD codes | 171.5 | 172 |
| Average number of incidences of ICD codes | 194.5 | 189.3 |
| Minimal number of incidences of ICD codes | 3 | 4 |
| Maximal number of incidences of ICD codes | 750 | 767 |
